# Examining the effects of pill counts on treatment retention in buprenorphine maintenance therapy

**DOI:** 10.1016/j.dadr.2026.100478

**Published:** 2026-09-10

**Authors:** Jack Larsen, Patrick Kenney, Tonya Milam, Joyce Troxler, Caleb Osborne, Maria Zambrano

**Affiliations:** Department of Family Medicine, East Tennessee State University Quillen College of Medicine, Johnson City, TN 37614, USA

**Keywords:** Pill counts, Medication callbacks, Buprenorphine, Medication adherence, misuse deterrent

## Abstract

**Introduction:**

Buprenorphine is internationally recognized as an effective treatment for Opioid Use Disorder (OUD). Pill counting, a quantitative assessment of medication supply to monitor prescription medication adherence, is often recommended to monitor buprenorphine adherence. However, there is little evidence to support pill counting or examine its impact on addiction treatment. This retrospective review seeks to examine retention in treatment before and after implementation of a novel pill count protocol in outpatient substance use treatment.

**Methods:**

This observational retrospective cohort study consisted of patients receiving buprenorphine or buprenorphine/naloxone for treatment of OUD as part of an outpatient university-affiliated substance use treatment program in the United States. Patients seen prior to the introduction of pill counts (n = 103) were compared to patients (n = 107) who had a pill count performed after the introduction of pill counts.

**Results:**

Retention in treatment was higher after pill count implementation, but the statistical evidence was not consistent across analyses. The incidence of an inappropriate pill count was 36.4%. No significant difference in retention in treatment was found between those with appropriate counts and inappropriate counts (94.8% versus 95.5%, p = 0.87). The most common clinician response to an inappropriate pill count was closer follow up (43.6%). We describe our pill count protocol as a prototype.

**Conclusions:**

The findings from this study show inconsistent statistical support for a positive association between buprenorphine pill counts and retention in OUD treatment. A prototype pill count may allow for standardization and validation of this commonly performed yet poorly understood practice.

## Highlights

• Pill counts may be used to monitor treatment adherence with buprenorphine.
• Pill counts did not adversely affect retention in treatment in our study.
• A buprenorphine pill count protocol can help standardize medication callbacks.
• Inappropriate pill counts do not significantly decrease retention in treatment.

## Graphical Abstract

**Figure:**
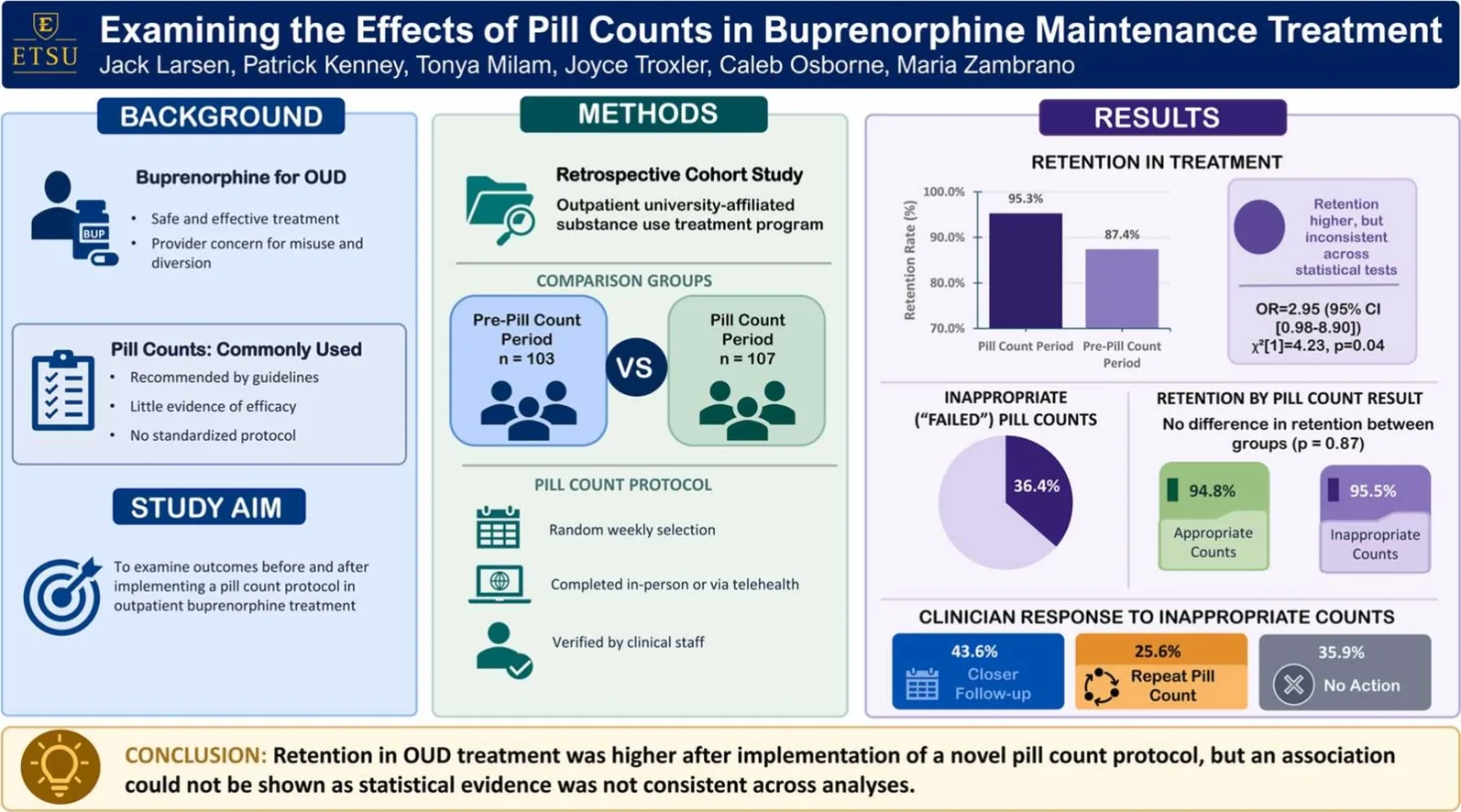

## 1 Introduction

Buprenorphine, methadone, and naltrexone are three commonly used and effective pharmacotherapies for the treatment of Opioid Use Disorder (OUD). OUD represents a global public health concern and was responsible for an estimated 99,556 deaths in 2021, reflecting a 17% increase since 2017 (Jiang et al., 2025, Zhang et al., 2025). In response, countries across the world have sought to expand access to evidence-based pharmacotherapy for OUD while implementing policies intended to balance medication access with the prevention of misuse and diversion (World Health Organization., 2024; Lofwall and Walsh, 2014). In the United States, 3.7% of adults aged ≥ 18 years needed treatment for OUD in 2022, yet only 25.1% of these received pharmacotherapy (Dowell et al., 2024). This has led to a call for increased access to pharmacotherapy, including buprenorphine, in the United States (Wakeman, 2025). Efforts have included the Medication Access and Expansion Act of 2023, which eliminated special prescribing requirements (the “X-waiver”) for providers. However, buprenorphine misuse, defined as using buprenorphine in a different way than as prescribed, remains a significant concern among prescribing providers (Lin et al., 2018). Misuse includes, but is not limited to, diversion, defined as unauthorized rerouting or misappropriation of prescribed buprenorphine (Lofwall and Walsh, 2014). Therefore, to increase provider comfort in providing medication access, it is important to address the risk of buprenorphine misuse, while also prioritizing the needs and experiences of unique patients engaged in treatment.

Aiming to mitigate misuse risk, multiple guidelines recommend the use of pill counts (Substance Abuse and Mental Health Service Administration, 2018, The ASAM National Practice Guideline for the Treatment of Opioid Use Disorder: 2020 Focused Update, 2020). Pill counts, or “medication callbacks,” are quantitative assessments of a patient’s current medication supply designed to monitor pharmacotherapy. Inappropriate pill counts can be defined as those where a patient did not present the correct quantity of prescription medication that would be expected based on its fill date and administration instructions. Adherence can be defined as the extent to which patients take medications as prescribed by their health care providers (Osterberg and Blaschke, 2005). It is important to note that inappropriate pill counts do not necessarily indicate poor adherence, treatment engagement, or medication misuse. Pill counts are often recommended as part of a universal precautions plan, which includes other interventions such as patient contracts, urine drug screening, observed dosing, frequent office visits, and government monitoring databases. However, there are no studies that have isolated the impact of pill counts on buprenorphine treatment outcomes. Thus, our study aimed to observe their effects on outpatient treatment retention rates. Also, although recommended by guidelines, little is known about actual provider practice implementation. A study in 2009 reported a mean average 4.4 safeguards taken to reduce the risk of diversion among providers prescribing buprenorphine (Yang et al., 2013). However, only 29% of the 2330 providers surveyed indicated using pill counts as one of these measures. Pill counts, in addition to the universal precautions mentioned above, may be particularly helpful for monitoring patients with an established history of poor adherence or medication misuse.

Pill counts are a practice largely inherited from the field of pain medicine, where they are often recommended as part of universal precautions for patients using prescription opioids (Manchikanti et al., 2017). However, a systematic review showed there was little evidence to support their use in opioid therapy for pain (Gill et al., 2022), highlighting a gap between common practice and supporting evidence. Similarly, there is also a lack of evidence regarding effectiveness of pill counting in OUD.

Although recommended uniformly, there are no widely accepted standardized protocols for buprenorphine pill counts, although an individual one was described in a previous study (Fareed et al., 2014). Patients in this study were selected randomly and contacted via phone and required to report in-person to their provider’s office within 24 h to have their medication counted. However, no other pill counting protocol descriptions were found in the literature or in the practice guidelines that recommend them. Thus, one of the primary goals of our team was to develop a novel protocol that can be used as a prototype for further research. Provider responses to pill count results are also heterogenous and poorly described in the literature, so these were identified as an important outcome of the protocol. Of note, there is also a growing attempt to incorporate telehealth capabilities into pill count protocols to complete buprenorphine pill counts remotely for patients with access barriers (Welsh, 2016, DeWorsop et al., 2016), so telehealth capabilities were also included in the protocol.

Rates of inappropriate buprenorphine pill counts in outpatient treatment are poorly described. A 2014 study found an inappropriate rate of 34.7%, however this was reported in aggregate with Urine Drug Screen (UDS) results (Fareed et al., 2014). No isolated inappropriate buprenorphine pill count rates were found on review of the literature. Rates of inappropriate pill counts have been better reported for other substance use disorder pharmacotherapies such as naltrexone, varenicline, and buspirone (Namkoong et al., 1999, Peng et al., 2018, McRae-Clark et al., 2015), but these studies involved non-controlled substance therapies that likely have less potential for misuse.

We also have concern that performing buprenorphine pill counts could negatively impact care, for example decreasing treatment retention rates, via prescription termination or even discharge. A 2018 survey of buprenorphine prescribers indicated prescribers performing higher percentages of pill counts reported greater willingness to terminate patients from treatment for diverting medication (Lin et al., 2018). About half of providers surveyed strongly agreed and another thirty percent agreed that they would terminate treatment when they suspect diversion. Termination could lead to lower rates of retention in treatment, which is used as a proxy for drug use improvement in the treatment of OUD (Zhang et al., 2003). In a population with whom engagement and trust are central to effective treatment, it is also possible pill counts may inadvertently introduce suspicion or create a punitive dynamic in a treatment environment.

There is a paucity of literature describing buprenorphine pill counts and examining their effects on treatment. Thus, this study was designed to: 1) examine the effect of implementing pill counts on retention in outpatient substance use treatment with buprenorphine, 2) describe our pill counting protocol as a prototype, 3) quantify inappropriate pill count rate, and 4) evaluate the responses taken by clinicians to inappropriate pill counts.

## 2 Methods

### 2.1 Sample

This observational retrospective cohort study consisted of patients receiving buprenorphine or buprenorphine/naloxone for treatment of OUD as part of an outpatient university-affiliated substance use treatment program in the United States. Patients seen between September and December 2024, prior to the introduction of pill counts, were identified by retrospective chart review as controls (n = 103). Intervention group patients (n = 107) had at least 1 pill count performed between January 2025 and January 2026. Patients not being treated with buprenorphine were excluded. This study was reviewed by the Institutional Review Board at East Tennessee State University and determined to qualify for exempt status due to its retrospective observational design involving existing clinical data. The requirement for informed consent was waived.

### 2.2 Study procedures

**Figure A.1:**
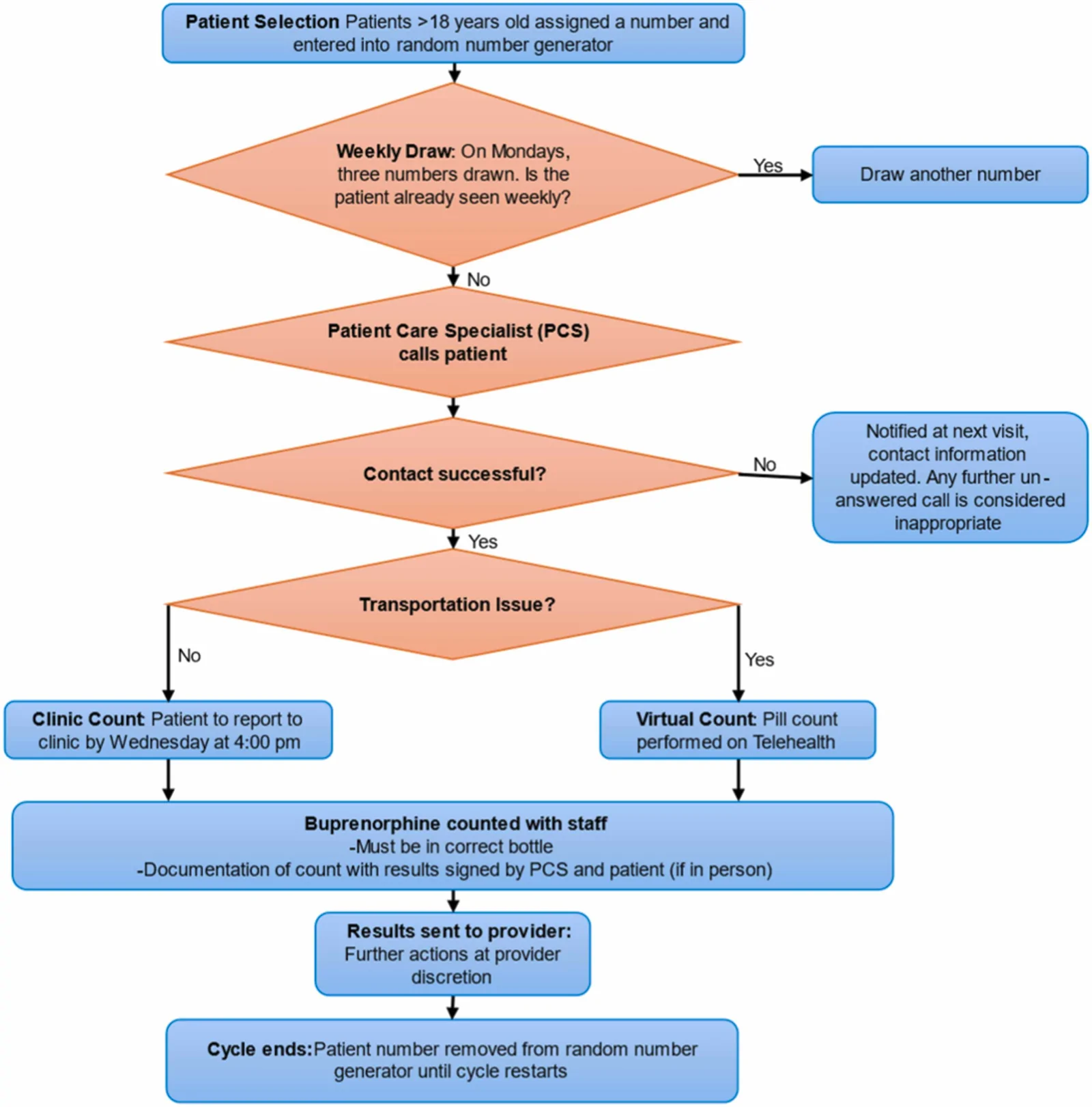
Pill Count Protocol. Patients ≥ 18 years were assigned a unique identifier and entered into a random number generator for weekly selection. Up to three patients were selected each Monday; if a selected patient had already been seen that week, an alternate number was drawn. Pill counts were conducted either in person or via telehealth depending on transportation barriers. All counts required verification by clinic staff, with documentation completed and results communicated to the prescribing provider for further clinical decision-making

Patients in the intervention group underwent random pill counts according to the protocol described in Fig. A.1, which was implemented in January 2025. All adult clinic patients prescribed buprenorphine were assigned a number and entered into a random number generator. Each week, three numbers were randomly drawn and the corresponding patients chosen for a pill count. A single Patient Care Specialist (PCS) was specially trained in the protocol via an educational session with study staff. Selected patients were called via telephone by the PCS and given two days to report to the clinic for a pill count. Upon arrival, the PCS validated the medication bottle and documented the number of tablets or films remaining. Validation included ensuring the medication was in its original dispensing container, with the patient’s name, prescribing provider’s name, and fill date appropriately verified on a standardized reporting form. An inappropriate pill count was recorded when a patient did not present the correct quantity of prescription medication that would be expected based on its fill date and administration instructions, pass validation process, or show for the appointment. The results of the count were sent to that patient’s medical provider for further action, which was determined at their clinical discretion. The patient’s number was then removed from the random number generator until all patients had been selected, at which point the cycle restarted. Patients already being seen weekly had their counts deferred until they achieved greater follow up intervals because they were determined to already have closer monitoring for medication adherence (more frequent drug testing, more frequent office visits, and shorter prescription intervals). Patients who were unable to be contacted were notified at their next visit, had their contact information updated, and were reentered into the number generator. Any further unanswered call was considered an inappropriate count- all patients are in our clinic are required to take part in the pill counts as part of their controlled substance contract, and it was felt it would be unfair to allow patients with communication limitations to skip the requirement, as no suitable alternative could be practically arranged. Patients with transportation challanges were given the option of digital verification with the PCS over video link. Non-random counts (for example, repeat counts in response to an initial inappropriate count) were not included to reduce bias.

Retrospective chart review was used to identify demographics and 90-day retention in treatment for patients in both groups, with retention in treatment measured 90 days after the pill count in the intervention group. In the intervention group, qualitative chart review was performed by a research staff member trained using predefined criteria to describe provider responses to inappropriate pill counts. These were required to be significant changes to the patient’s treatment plan that were documented in the electronic health record, or an absence of this documentation. Subgroup analysis was also performed within the intervention group to compare rates of treatment retention between patients with appropriate versus inappropriate pill counts.

### 2.3 Statistical analysis

Descriptive statistics were used to summarize baseline demographic and clinical characteristics for both control and intervention groups. Categorical variables were reported as frequencies and percentages and compared using Pearson chi-squared (χ²) tests to assess for baseline differences between groups. Retention rates were analyzed by comparing proportions between groups using Pearson chi-squared testing. Given our modest sample size and the potential for small expected call counts, Yates’ continuity correction and Fischer’s exact tests were employed as sensitivity analyses when appropriate.

Effect size was estimated using odds ratios (OR) with corresponding 95% confidence intervals (CI), calculated from 2 × 2 contingency tables, and subgroup analysis within the intervention group was conducted using Pearson chi-squared testing to compare retention rates between patients with appropriate versus inappropriate pill counts.

All statistical tests were two-sided, and statistical significance was defined as p < 0.05.

## 3 Results

### 3.1 Sample description

Table A.1 shows the demographic characteristics of patients in the study. There were no significant differences between demographic variables in the control (n = 103) and intervention (n = 107) groups. Subjects were more likely to be female in both control (68.9%) and intervention (62.6%) groups. Both groups were primarily White in ethnicity (98.0% for control group and 96.2% for intervention group). Medicaid was the most common insurance coverage in both control and intervention groups (48.5% and 40.2%, respectively). Most patients were between ages 25 and 44 (66.0% for control group and 67.2% for intervention group).

### 3.2 The relationship between pill counts and retention in treatment

There was higher retention in treatment after implementation of pill counts compared with control using Pearson χ² analysis (95.3% vs. 87.4%; χ²[1]=4.23, p = 0.04). This finding approached but did not consistently meet statistical significance across sensitivity analyses, including a borderline Fisher’s exact test result (p = 0.049) and a non-significant Yates-corrected χ² analysis (χ²=3.28, p = 0.07). The estimated odds ratio was 2.95 (95% CI 0.98–8.90).

### 3.3 Inappropriate pill counts and retention in treatment

The frequency of inappropriate pill counts was 36.4%. Of the 39 patients with inappropriate pill counts, 13 (33.3%) were due to nonresponse to multiple voicemails left and 6 (15.4%) were due to a disconnected/nonviable phone number, even after their contact information was updated per the protocol. In subgroup analysis, rates of treatment retention were not significantly different for patients who had inappropriate pill counts as compared to patients with appropriate pill counts (94.8% versus 95.6%, χ ² [1]=0.03, p = 0.87).

### 3.4 Provider responses to inappropriate pill counts

Among the 39 documented inappropriate pill counts, the most common provider response was closer follow up/shorter prescription length (43.6%). The next most common response was a repeat pill count (25.6%), which was appropriate in all but one case. A significant proportion of inappropriate pill counts (35.9%) resulted in no provider response discernable by chart review. Other less common provider responses were decreasing buprenorphine dose (1 patient), switching to a long-acting injectable formulation (1 patient), and having a family member administer the medication (1 patient).

## 4 Discussion

Pill counting is a commonly recommended and used method of determining adherence to buprenorphine for the treatment of OUD, yet protocols are scarcely described, and little is known about its effects on treatment outcomes. Retention in treatment was higher after pill count implementation, but the statistical evidence was not consistent across analyses, so the finding should be interpreted cautiously rather than as either a definite association or no association. This is impactful because pill counting is commonly used, and retention in treatment is associated with improved control of OUD (Zhang et al., 2003). It is reassuring to see no decrease in retention in treatment after implementation of pill counts.

Pill counting may have beneficial effects on treatment. Placing verification of adherence in the patient’s hands may attenuate feelings of distrust between the provider and patient, and thus make patients more likely to remain in treatment after an appropriate count. Conversely, inappropriate counts and their consequences may serve as negative reinforcers to improve treatment program adherence.

However, there is also some concern that pill counts may have negative effects on treatment. Buprenorphine prescribers with greater percentages of patients undergoing pill counts report greater willingness to terminate patients from treatment for diversion (Lin et al., 2018). Because buprenorphine is a controlled substance, practitioners with good intentions may fear legal repercussions of continuing to prescribe it after an inappropriate pill count. Also, because reasons for inappropriate pill counts are heterogenous, it is hard to establish direct correlation between diversion and an inappropriate pill count. As noted in Substance Abuse and Mental Health Services Administration guidelines, diversion as a behavior may be due to uncontrolled symptoms of substance use disorder, and this behavior warrants a change in a patient’s treatment plan (Substance Abuse and Mental Health Service Administration, 2018). This may or may not include termination in pharmacotherapy, but could also include closer follow-up or alternate pharmacotherapy, which may instead improve retention in a treatment program. Additionally, it is possible pill counts themselves may contribute to medication misuse or decrease adherence. For example, a patient who anticipates a pill count could alter their medication use or seek additional medication from other sources in order to satisfy the requirements. Inappropriate pill count results may also foster feelings of mistrust between a patient and a treatment program. This may shift patient motivation from an intrinsic health focus to a fear-based impetus centered on avoidance of punitive treatment withdrawal for perceived noncompliance. It is, however, reassuring to see that inappropriate pill counts did not result in significantly decreased retention in treatment relative to patients with appropriate pill counts.

Inappropriate pill counts were common in this study (36.4%), consistent with rates described elsewhere (34.7%, Fareed et al., 2014). There are a multitude of reasons a patient might have an inappropriate pill count, but these do not necessarily indicate adherence or misuse (Osterberg and Blaschke, 2005). An inappropriately low supply could be due to medication overuse. Cravings and withdrawal symptoms may cause patients to take more buprenorphine than prescribed due to inadequate buprenorphine dosing, indicating a need for increased dosing. Patients may also forget to bring in their medication in its entirety or discard unused doses, resulting in inappropriately low counts. Illicit causes include theft, sharing or selling medication (diversion). An excessively high supply may indicate medication underuse or cognitive dysfunction. Another common reason for an inappropriate pill count is communication breakdown. Significant numbers of inappropriate pill counts resulted from disconnected phone numbers or unanswered voicemails, in a patient population that faces significant socioeconomic barriers to healthcare. Although our protocol attempted to accommodate for this by allowing a repeat contact after updating of contact information, a significant number of patients still were not able to be contacted the second time. Therefore, even accommodations such as these may not remedy the socioeconomic communication challenges patients with substance use disorder struggle with, such as homelessness. Additionally, pill counting alone cannot account for use from other sources, such as prior surpluses, doses procured through another provider, or diversion. Interestingly, patients with inappropriate buprenorphine pill counts have been shown to often still have therapeutic levels of buprenorphine/norbuprenorphine in their system (Fareed et al., 2014). Thus, there is no guaranteed relationship between the number of pills remaining and the patient’s actual use of the medication (Gill et al., 2022). Pill counting also requires expenditure of finite resources (clinic staff and provider time) that could be directed elsewhere.

Provider responses to inappropriate pill counts were highly varied. Although desirable for consistency, it was felt unfeasible to standardize provider responses in the pill count protocol due to the heterogenous nature of the patient population and varied reasons for inappropriate counts. For example, a patient who is missing pill counts due to telephone access issues likely warrants different treatment than a patient with inappropriately low medication supply and drug screens persistently negative for prescribed buprenorphine. The relatively high rate of no provider action being taken in response to an inappropriate count may point towards a need for better provider education or documentation habits. These instances may also represent neutral clinical decisions that were not documented or missed opportunities for intervention. No patients had their prescriptions terminated as a direct result of pill counts, but it seems logical this action may make patients more likely to leave a treatment program, and may occur more frequently at other practices. Long-Acting Injectable (LAI) formulations of buprenorphine may provide an attractive alternative for patients with repeated inappropriate counts. Closer follow-up for patients with inappropriate pill counts seems prudent, as it decreases the supply of medication that could potentially be misused with each prescription. These patients also may warrant closer follow-up as they have been shown to have more comorbid psychiatric illness, and be more likely to test positive for benzodiazepines or cannabis during treatment (Fareed et al., 2014). However, the potential benefits of closer monitoring must be balanced with the risks of damaging trust in the therapeutic relationship and decreasing patient engagement in treatment. Lockbox distribution may also provide protection against medication theft (Grist et al., 2023).

Telehealth pill counts are an attractive solution for patients with transportation barriers. Our protocol allowed for such, and others have noted their utility (Welsh, 2016). However, telehealth pill counts do not circumvent many of the accuracy concerns and communications barriers previously described.

Limitations of this study include a relatively small sample size. We also did not correlate pill count results with other measures of patient adherence, such as urine drug screens. This limits the extent to which the current findings can correlate with other measures of medication adherence. Patients already being seen on a weekly basis had their counts deferred until they were being seen less frequently and therefore likely achieved a greater degree of stability. Patients in the pre-intervention group were included regardless of follow up interval, which may have introduced bias into the sample. Patient history, time influences, and prescriber differences may have also influenced results. The study population was disproportionately White and female, which may limit generalizability to other patient populations. The clinic moved physical locations several months after implementing the pill count protocol, which could have influenced results as some of the later counts were performed at a different physical location.

Overall, buprenorphine pill counting is a not-well understood tool that may be cautiously used as a part of a universal precautions plan intended to reduce medication misuse, in conjunction with other tools such as controlled substance contracts, drug testing, lockbox provision, and government prescription monitoring programs. While buprenorphine pill counts could provide the benefit of monitoring medication adherence, there is not evidence that they actually increase adherence and providers need to be aware that they may have unintended consequences on patients in substance use treatment. Our study indicates implementation of a pill count protocol may have an association with retention in outpatient treatment using buprenorphine, but needs to be interpreted cautiously due to inconsistent statistical evidence. Our findings also indicate that the results of a pill count result do not appear to be associated with retention in treatment. Pill count protocols, including provider responses to inappropriate pill counts, are heterogenous, and the establishment of a standard protocol could streamline patient care as well as further research.

Future research is recommended to investigate the effects of buprenorphine pill counting on outcomes in substance use treatment, including association with other measures of adherence, such as drug screen results. We also encourage replication of our research, including validation of our protocol and publishing other treatment centers’ pill count protocols, which could allow for comparison and eventual standardization of a process recommended across multiple practice guidelines.

## CRediT authorship contribution statement

**Jack Larsen:** Writing – review & editing, Writing – original draft, Supervision, Project administration, Methodology, Data curation, Conceptualization. **Patrick Kenney:** Writing – review & editing, Writing – original draft, Resources, Methodology, Formal analysis, Conceptualization. **Maria Zambrano:** Writing – review & editing, Data curation. **Caleb Osborne:** Writing – review & editing, Data curation. **Joyce Troxler:** Writing – review & editing, Methodology, Data curation, Conceptualization. **Tonya Milam:** Methodology, Data curation, Conceptualization.

## Ethics declaration

The authors have NOT obtained informed consent from participants or their legal representatives. This was retrospective in nature.

This study was performed in compliance with relevant laws, regulatory frameworks and guidelines where the research took place. The ETSU QCOM IRB granted an exemption. The authors provided the following explanation: his study was reviewed by the Institutional Review Board at East Tennessee State University and determined to qualify for exempt status due to its retrospective observational design involving existing clinical data. The requirement for informed consent was waived.

## Funding

This research did not receive any specific grant from funding agencies in the public, commercial, or not-for-profit sectors.

## Declaration of Competing Interest

The authors declare that they have no known competing financial interests or personal relationships that could have appeared to influence the work reported in this paper.

## Figures and Tables

**Table A.1:** Demographic and Clinical Characteristics of Study Participants by Group

**Characteristic**	**Controls (N = 103)**	**Intervention (N = 107)**
**Age, n (%)**		
18–24	5 (4.9%)	3 (2.8%)
25–44	68 (66.0%)	72 (67.3%)
45–64	26 (25.2%)	27 (25.2%)
≥ 65	4 (3.9%)	5 (4.7%)
**Race/Ethnicity, n (%)**		
White	101 (98.1%)	103 (96.3%)
African American	1 (1.0%)	2 (1.9%)
Latino	1 (1.0%)	2 (1.9%)
Asian	0 (0.0%)	0 (0.0%)
Other	0 (0.0%)	0 (0.0%)
**Sex, n (%)**		
Male	32 (31.1%)	40 (37.4%)
Female	71 (68.9%)	67 (62.6%)
**Insurance Status, n (%)**		
Private	21 (20.4%)	28 (26.2%)
Medicare	14 (13.6%)	18 (16.8%)
Medicaid	50 (48.5%)	43 (40.2%)
Uninsured/Self-pay	18 (17.5%)	18 (16.8%)

Baseline characteristics were generally comparable between the control (N = 103) and

intervention (N = 107) groups, with similar distributions across age, sex, race/ethnicity, and

insurance status. The majority of participants in both groups were aged 25–44 years and

identified as White, and Medicaid was the most common primary payer. Minor differences

were observed, including a slightly higher proportion of male and privately insured

participants in the intervention group.
